# Geographic Differences in COVID-19 Cases, Deaths, and Incidence — United States, February 12–April 7, 2020

**DOI:** 10.15585/mmwr.mm6915e4

**Published:** 2020-04-17

**Authors:** CDC COVID-19 Response Team, CDC COVID-19 Response Team, Stephanie Bialek, Virginia Bowen, Nancy Chow, Aaron Curns, Ryan Gierke, Aron Hall, Michelle Hughes, Tamara Pilishvili, Matthew Ritchey, Katherine Roguski, Benjamin Silk, Tami Skoff, Preethi Sundararaman, Emily Ussery, Michael Vasser, Hilary Whitham, John Wen

**Affiliations:** All authors have completed and submitted the International Committee of Medical Journal Editors form for disclosure of potential conflicts of interest. No potential conflicts of interest were disclosed.; CDC; CDC; CDC; CDC; CDC; CDC; CDC; CDC; CDC; CDC; CDC; CDC; CDC; CDC; CDC; CDC; CDC.

*On April 10, 2020, this report was posted online as an *MMWR *Early Release.*

Community transmission of coronavirus disease 2019 (COVID-19) was first detected in the United States in February 2020. By mid-March, all 50 states, the District of Columbia (DC), New York City (NYC), and four U.S. territories had reported cases of COVID-19. This report describes the geographic distribution of laboratory-confirmed COVID-19 cases and related deaths reported by each U.S. state, each territory and freely associated state,* DC, and NYC during February 12–April 7, 2020, and estimates cumulative incidence for each jurisdiction. In addition, it projects the jurisdiction-level trajectory of this pandemic by estimating case doubling times on April 7 and changes in cumulative incidence during the most recent 7-day period (March 31–April 7). As of April 7, 2020, a total of 395,926 cases of COVID-19, including 12,757 related deaths, were reported in the United States. Cumulative COVID-19 incidence varied substantially by jurisdiction, ranging from 20.6 cases per 100,000 in Minnesota to 915.3 in NYC. On April 7, national case doubling time was approximately 6.5 days, although this ranged from 5.5 to 8.0 days in the 10 jurisdictions reporting the most cases. Absolute change in cumulative incidence during March 31–April 7 also varied widely, ranging from an increase of 8.3 cases per 100,000 in Minnesota to 418.0 in NYC. Geographic differences in numbers of COVID-19 cases and deaths, cumulative incidence, and changes in incidence likely reflect a combination of jurisdiction-specific epidemiologic and population-level factors, including 1) the timing of COVID-19 introductions; 2) population density; 3) age distribution and prevalence of underlying medical conditions among COVID-19 patients (*1*–*3*); 4) the timing and extent of community mitigation measures; 5) diagnostic testing capacity; and 6) public health reporting practices. Monitoring jurisdiction-level numbers of COVID-19 cases, deaths, and changes in incidence is critical for understanding community risk and making decisions about community mitigation, including social distancing, and strategic health care resource allocation.

This analysis includes all laboratory-confirmed COVID-19 cases^†^ reported to CDC during February 12–April 7 from health departments in all 50 U.S. states, eight U.S. territories and freely associated states, DC, and NYC. Beginning on March 3, jurisdictions reported aggregate numbers of cases and deaths daily. Cases and deaths reported by the state of New York are exclusive of those reported by NYC. National and jurisdiction-specific case doubling times for the 10 jurisdictions with the most cases were estimated for April 7 by calculating the number of days before April 7 in which the observed cases were equal to half that reported on April 7. National and jurisdiction-specific cumulative incidences were estimated using 2018 population estimates.^§^ Absolute 7-day changes in cumulative incidence were calculated by subtracting the jurisdiction-specific cumulative incidence on March 31 from that observed on April 7.

As of April 7, a total of 395,926 COVID-19 cases were reported in the United States (Table). Cases were reported by all 50 states, DC, NYC, Guam, the Northern Mariana Islands, Puerto Rico, and the U.S. Virgin Islands. Two thirds of all COVID-19 cases (66.7%) were reported by eight jurisdictions: NYC (76,876), New York (61,897), New Jersey (44,416), Michigan (18,970), Louisiana (16,284), California (15,865), Massachusetts (15,202), and Pennsylvania (14,559) (Figure 1). The overall cumulative COVID-19 incidence in the United States was 119.6 cases per 100,000 population on April 7 (Table). Among jurisdictions in the continental United States, cumulative incidence was lowest in Minnesota (20.6) and highest in NYC (915.3). Nine reporting jurisdictions had rates above the national rate: NYC (915.3), New York (555.5), New Jersey (498.6), Louisiana (349.4), Massachusetts (220.3), Connecticut (217.8), Michigan (189.8), DC (172.4), and Rhode Island (133.7).

**TABLE:** Reported COVID-19 cases and deaths and estimated cumulative incidence,* March 31 and April 7, 2020, and change in cumulative incidence from March 31 to April 7, 2020 — U.S. jurisdictions

Jurisdiction	March 31		April 7			March 31–April 7
	No. of cases	Cumulative incidence*	No. of cases	No. (%) of deaths	Cumulative incidence*	Absolute change in cumulative incidence*
**States, District of Columbia, and New York City**						
Alabama	999	20.4	2,197	39 (1.8)	44.9	24.5
Alaska	133	18.0	213	6 (2.8)	28.9	10.8
Arizona	1,289	18.0	2,575	73 (2.8)	35.9	17.9
Arkansas	560	18.6	993	18 (1.8)	32.9	14.4
California	8,131	20.6	15,865	374 (2.4)	40.1	19.6
Colorado	2,966	52.1	5,429	179 (3.3)	95.3	43.2
Connecticut	3,128	87.6	7,781	277 (3.6)	217.8	130.2
Delaware	319	33.0	928	16 (1.7)	95.9	63.0
District of Columbia	495	70.5	1,211	24 (2.0)	172.4	101.9
Florida	6,490	30.5	14,302	296 (2.1)	67.1	36.7
Georgia	4,585	43.6	9,713	351 (3.6)	92.3	48.7
Hawaii	185	13.0	362	5 (1.4)	25.5	12.5
Idaho	525	29.9	1,210	15 (1.2)	69.0	39.0
Illinois	5,994	47.0	13,549	380 (2.8)	106.3	59.3
Indiana	2,159	32.3	5,507	173 (3.1)	82.3	50.0
Iowa	497	15.7	1,048	26 (2.5)	33.2	17.5
Kansas	428	14.7	900	27 (3.0)	30.9	16.2
Kentucky	591	13.2	1,149	65 (5.7)	25.7	12.5
Louisiana	5,237	112.4	16,284	582 (3.6)	349.4	237.1
Maine	303	22.6	519	12 (2.3)	38.8	16.1
Maryland	1,660	27.5	5,529	124 (2.2)	91.5	64.0
Massachusetts	6,620	95.9	15,202	356 (2.3)	220.3	124.3
Michigan	7,615	76.2	18,970	845 (4.5)	189.8	113.6
Minnesota	689	12.3	1,154	39 (3.4)	20.6	8.3
Mississippi	1,073	35.9	2,003	67 (3.3)	67.1	31.1
Missouri	1,327	21.7	3,037	53 (1.7)	49.6	27.9
Montana	203	19.1	332	6 (1.8)	31.3	12.1
Nebraska	177	9.2	478	10 (2.1)	24.8	15.6
Nevada	1,113	36.7	2,087	71 (3.4)	68.8	32.1
New Hampshire	367	27.1	747	13 (1.7)	55.1	28.0
New Jersey	18,696	209.9	44,416	1,232 (2.8)	498.6	288.7
New Mexico	315	15.0	794	13 (1.6)	37.9	22.9
New York^†^	32,656	293.1	61,897	1,378 (2.2)	555.5	262.4
New York City	41,771	497.3	76,876	4,111 (5.3)	915.3	418.0
North Carolina	1,584	15.3	3,221	46 (1.4)	31.0	15.8
North Dakota	126	16.6	237	4 (1.7)	31.2	14.6
Ohio	2,199	18.8	4,782	167 (3.5)	40.9	22.1
Oklahoma	565	14.3	1,472	67 (4.6)	37.3	23.0
Oregon	690	16.5	1,181	33 (2.8)	28.2	11.7
Pennsylvania	4,843	37.8	14,559	240 (1.6)	113.7	75.9
Rhode Island	520	49.2	1,414	30 (2.1)	133.7	84.6
South Carolina	1,083	21.3	2,417	51 (2.1)	47.5	26.2
South Dakota	108	12.2	320	6 (1.9)	36.3	24.0
Tennessee	2,239	33.1	4,139	72 (1.7)	61.1	28.1
Texas	3,266	11.4	8,262	154 (1.9)	28.8	17.4
Utah	934	29.5	1,804	13 (0.7)	57.1	27.5
Vermont	293	46.8	575	23 (4.0)	91.8	45.0
Virginia	1,484	17.4	3,645	75 (2.1)	42.8	25.4
Washington	4,896	65.0	8,682	394 (4.5)	115.2	50.2
West Virginia	162	9.0	412	4 (1.0)	22.8	13.8
Wisconsin	1,351	23.2	2,578	92 (3.6)	44.3	21.1
Wyoming	120	20.8	221	0 (—)	38.3	17.5
**Territories and freely associated states**						
American Samoa	0	0.0	0	0 (—)	0.0	0.0
Federated States of Micronesia	0	0.0	0	0 (—)	0.0	0.0
Guam	71	42.8	122	4 (3.3)	73.6	30.8
Marshall Islands	0	0.0	0	0 (—)	0.0	0.0
Northern Mariana Islands	2	3.5	8	2 (25.0)	14.1	10.5
Palau	0	0.0	0	0 (—)	0.0	0.0
Puerto Rico	239	7.5	573	23 (4.0)	17.9	10.5
U.S. Virgin Islands	30	28.0	45	1 (2.2)	42.1	14.0
**U.S. Total**	**186,101**	**56.2**	**395,926**	**12,757 (3.2)**	**119.6**	**63.4**

* Cases per 100,000 population.

^†^ Excludes New York City.

**FIGURE 1:**
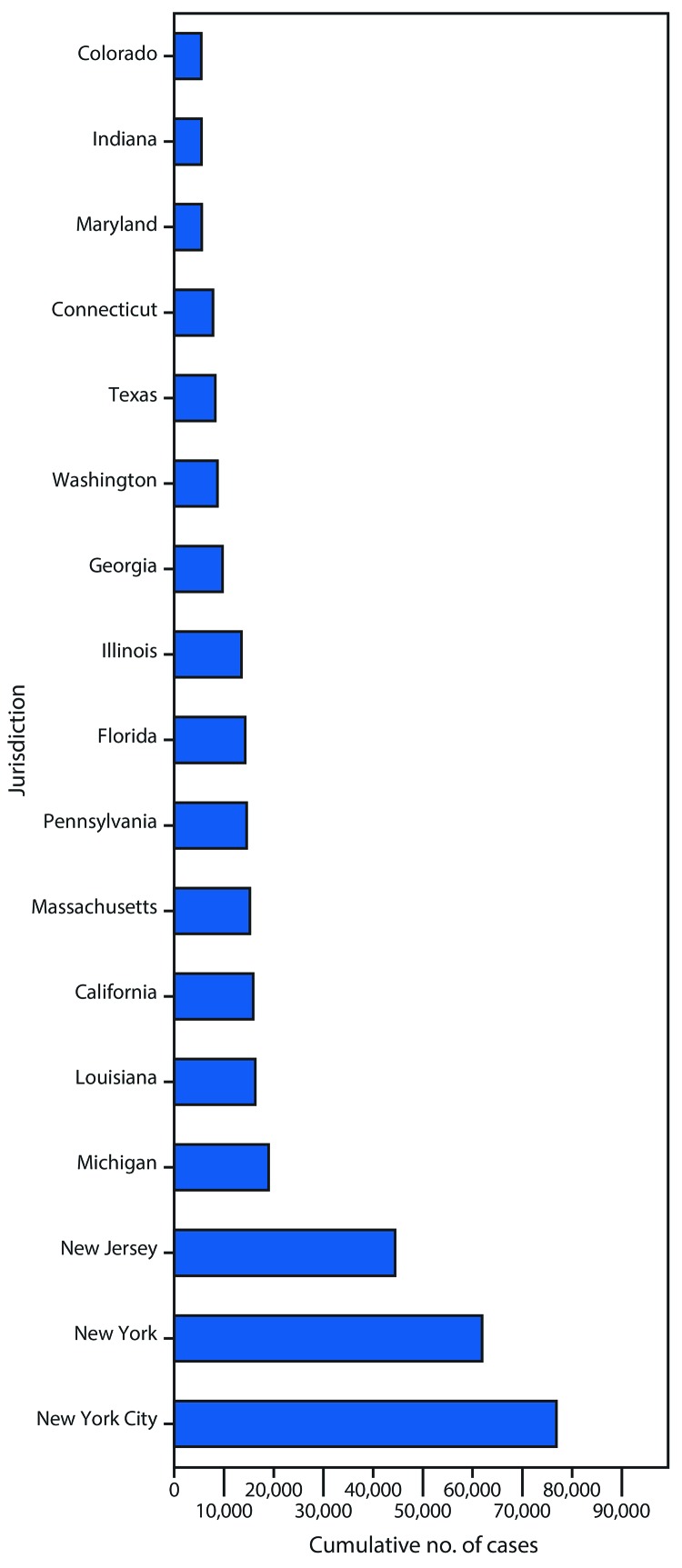
Cumulative number of reported COVID-19 cases, by jurisdiction — selected U.S. jurisdictions,*^,†^ April 7, 2020 **Abbreviation:** COVID-19 = coronavirus disease 2019 * Restricted to U.S. reporting jurisdictions with ≥5,000 COVID-19 cases reported as of April 7, 2020. ^†^ Data from New York are exclusive of New York City.

On April 7, nationwide case doubling time was approximately 6.5 days. Among the 10 jurisdictions reporting the most cases, doubling time ranged from 5.5 days in Louisiana to 8.0 days in NYC. During March 31–April 7, the overall cumulative incidence of COVID-19 increased by 63.4 cases per 100,000 (Table). This increase ranged from 8.3 in Minnesota to 418.0 in NYC. During the 7-day period, increases in 11 jurisdictions exceeded the national increase: NYC (418.0), New Jersey (288.7), New York (262.4), Louisiana (237.1), Connecticut (130.2), Massachusetts (124.3), Michigan (113.6), DC (101.9), Rhode Island (84.6), Pennsylvania (75.9), and Maryland (64.0) (Figure 2).

**FIGURE 2:**
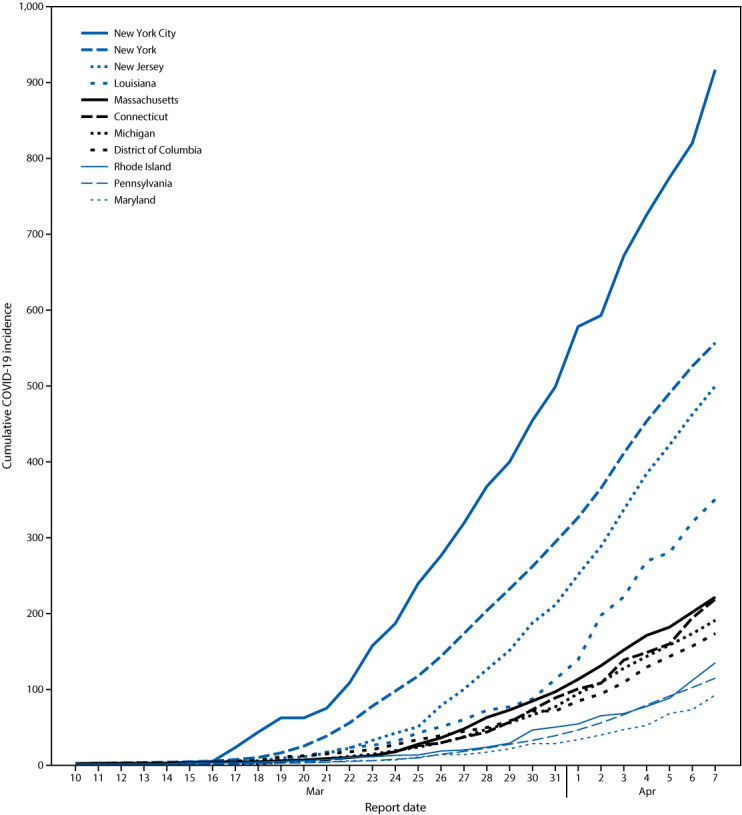
Cumulative incidence* of COVID-19, by report date — selected U.S. jurisdictions,^†^^,^^§^ March 10–April 7, 2020 **Abbreviation:** COVID-19 = coronavirus disease 2019. * Cases per 100,000 population. ^†^ Restricted to the 11 jurisdictions reporting the largest absolute increase in COVID-19 cumulative incidence during the most recent 7-day reporting period, March 31–April 7, 2020. ^§^ Data from New York are exclusive of New York City.

By April 7, 55 (98.2%) of the 56 jurisdictions reporting COVID-19 cases also reported at least one related death (Table); however, approximately half (52.7%) of all deaths (12,757) were reported from three jurisdictions: NYC (4,111), New York (1,378), and New Jersey (1,232) (Figure 3). Other jurisdictions reporting ≥300 deaths included Michigan (845), Louisiana (582), Washington (394), Illinois (380), California (374), Massachusetts (356), and Georgia (351). Case-fatality ratios ranged from 0.7% in Utah to 5.7% in Kentucky.

**FIGURE 3:**
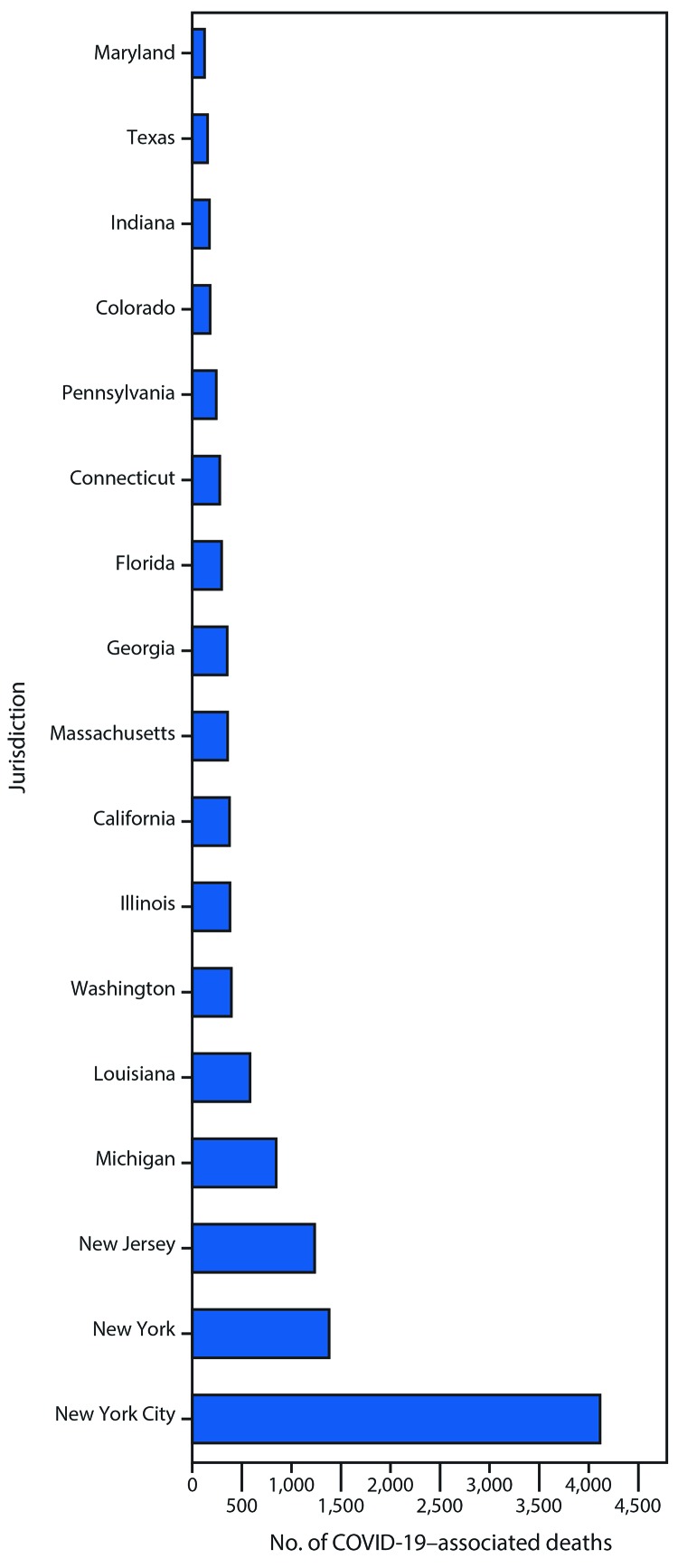
Number of reported COVID-19–related deaths, by jurisdiction — selected U.S. jurisdictions,*^,†^ April 7, 2020 **Abbreviation**: COVID-19 = coronavirus disease 2019. * Restricted to U.S. reporting jurisdictions with ≥5,000 COVID-19 cases reported as of April 7, 2020. ^†^ Data from New York are exclusive of New York City.

## Discussion

As of April 7, 2020, a total of 395,926 COVID-19 cases, including 12,757 deaths, were reported in the United States. The national cumulative incidence of 119.6 COVID-19 cases per 100,000 obscures significant geographic variation across reporting jurisdictions, with cumulative incidence in the continental U.S. ranging from 20.6 to 915.3 cases per 100,000. Increases in cumulative incidence during the most recent 7-day period (March 31–April 7) also varied widely, from 8.3 to 418.0 cases per 100,000. Geographic variation in numbers of COVID-19 cases and deaths, cumulative incidence, and changes in cumulative incidence likely reflects differences in epidemiologic and population factors as well as clinical and public health practices.

Differences in the timing of introduction and early transmission of SARS-CoV-2 (the virus that causes COVID-19) across jurisdictions might explain some of the observed geographic variation. The first documented U.S. cases of COVID-19 were among travelers returning from China and their immediate household contacts (*4*). During the third week of February, California, Oregon, and Washington reported the first U.S. cases with no known travel to China or exposure to a person with confirmed COVID-19. Case investigations indicated community transmission in these jurisdictions. Although one case of COVID-19 with an unknown exposure was reported during the fourth week of February in Florida, other cases with unknown exposure (i.e., community transmission) were not widely reported elsewhere until early March.

Because COVID-19 is primarily transmitted by respiratory droplets, population density might also play a significant role in the acceleration of transmission. Cumulative incidence in urban areas like NYC and DC exceeds the national average. Louisiana, which experienced a temporarily high population density because of an influx of visitors during Mardi Gras celebrations in mid-February, has a higher cumulative incidence and greater increase in cumulative incidence than other states in the South. Mardi Gras, which concluded on February 25, occurred at a time when cancelling mass gatherings (e.g., festivals, conferences, and sporting events) was not yet common in the United States.^¶^

The differential implementation and timing of community mitigation strategies across jurisdictions might have contributed to observed variation in incidence and changing incidences in this analysis. Community mitigation strategies, including school and workplace closures, cancellation of mass gatherings, and shelter-in-place orders, are recommended public health practices to reduce transmission during pandemics (*5*). COVID-19 modeling estimates suggest that mitigation could lead to substantial reductions in rates of infection, hospitalization, critical care, and death in North America (*6*). The effectiveness of these strategies to mitigate rates of infection and poor outcomes relies on their timely implementation before high levels of community transmission have been observed (*7*,*8*).

Differences in the availability of and approaches to SARS-CoV-2 testing, including testing patients across the spectrum of illness severity, likely contribute to geographic differences in COVID-19 incidence across jurisdictions. For example, the state of New York (excluding NYC) reported administering 4.9 tests per 1,000 population, which was higher than the national average of 1.6 (CDC, unpublished data, March 25, 2020); this expanded level of testing might have contributed to better ascertainment of cases and might partially explain the state’s higher case count and cumulative incidence. Jurisdictions that expanded public health and commercial laboratory testing later in March might also observe increases in cases and incidence as testing expands.

Differences in the numbers of deaths across jurisdictions might reflect the degree to which COVID-19 has been introduced into populations at high risk for severe outcomes (e.g., older adults or those with a high prevalence of underlying medical conditions). In Washington, which reported rapid spread of COVID-19 in several skilled nursing and long-term care facilities (*2*,*9*), the high number of deaths observed (394 [4.5%] among 8,682 cases) partially reflects the age and underlying medical conditions of populations affected by the outbreak (*1*,*3*). Geographic differences in reported case-fatality ratios might also reflect differences in testing practices; jurisdictions with relatively high proportions of deaths might be those where testing has been more limited and restricted to the most severely ill.

The findings in this report are subject to at least three limitations. First, reported COVID-19 cases are likely underestimated because of incomplete detection of cases and delays in case reporting. Reported deaths are also likely underestimated because of incomplete follow-up on all reported COVID-19 cases as well as death among persons infected with SARS-CoV-2 who did not receive a COVID-19 diagnosis. Second, the degree to which cases might go undetected or unreported varies across jurisdictions and might contribute significantly to the geographic variation observed in this analysis. Jurisdiction-level testing practices differ widely, and rapid increases in COVID-19 case detection have placed a high demand on health department infrastructure, leading to differential delays in case reporting. Finally, estimates of incidence, case-fatality ratios, and changes in incidence at the state and territorial levels might not be directly comparable across jurisdictions; further, COVID-19 “hotspots” and the effects of community mitigation efforts occurring within smaller geographic areas might be muted at this higher level of analysis.

Approximately 396,000 COVID-19 cases and 12,800 related deaths were reported in the United States as of April 7. The nation’s 60 reporting jurisdictions are experiencing various levels of COVID-19 transmission, resulting in substantial geographic differences in numbers of cases and deaths, incidence, and changes in incidence. Monitoring changes in numbers of reported cases and disease incidence within jurisdictions over time is critical to understanding and responding to the evolving local epidemiology of this outbreak. A clear picture of the magnitude and changing incidence within a jurisdiction will inform decisions regarding implementation of community mitigation strategies, including social distancing, and strategic allocation of human and capital resources, such as those supporting the health care infrastructure.

**Summary**

**What is already known about this topic?**

Community transmission of COVID-19 was first detected in the United States in February 2020. By mid-March, all 50 states, the District of Columbia, New York City, and four U.S. territories had reported cases of COVID-19.

**What is added by this report?**

As of April 7, cumulative incidence of COVID-19 ranged widely across U.S. jurisdictions (from 20.6 to 915.3 cases per 100,000) and 7-day increases in incidence varied considerably (from 8.3 to 418.0). This report highlights geographic differences in cases, deaths, incidence, and changing incidence.

**What are the implications for public health practice?**

Monitoring jurisdiction-level numbers of COVID-19 cases, deaths, and changes in incidence is critical for understanding community risk and making decisions about community mitigation, including social distancing, and strategic health care resource allocation.

## Footnotes

* American Samoa, Federated States of Micronesia, Guam, Marshall Islands, Northern Mariana Islands, Palau, Puerto Rico, and U.S. Virgin Islands.

† Laboratory-confirmed cases include those cases for which SARS-CoV-2, the virus that causes COVID-19, was detected by reverse transcription–polymerase chain reaction (RT-PCR) testing at a commercial, public health, or CDC laboratory. Cases among persons repatriated to the United States on State Department–chartered flights were excluded.

§ 2018 population estimates for the U.S. states, DC, and Puerto Rico were obtained from the U.S. Census Bureau (https://www.census.gov/data/tables/time-series/demo/popest/2010s-state-total.html); 2018 population estimates for NYC were obtained from the U.S. Census State Population Totals and Components of Change: 2010–2019 tables (https://www.census.gov/data/tables/time-series/demo/popest/2010s-state-total.html) and were subtracted from New York State estimates; the remaining jurisdictions’ estimates were obtained from The World Bank (https://data.worldbank.org/).

¶ CDC updated its interim guidance on COVID-19 on March 12, 2020, to reflect considerations for postponing or cancelling mass gatherings (e.g., >250 persons). https://www.cdc.gov/coronavirus/2019-ncov/community/large-events/mass-gatherings-ready-for-covid-19.html.
